# A37 POSTBIOTIC BACTERIAL MEMBRANE VESICLES FROM *B. SUBTILIS* DELIVER NOD2 LIGANDS AND PROMOTE IN VITRO WOUND RE-EPITHLIALIZATION THROUGH RIPK2 SIGNALING

**DOI:** 10.1093/jcag/gwad061.037

**Published:** 2024-02-14

**Authors:** L Baerg, D Philpott, R Mahadevan

**Affiliations:** . University of Toronto Institute of Biomedical Engineering, Toronto, ON, Canada; . University of Toronto Department of Immunology, Toronto, ON, Canada; . University of Toronto Department of Chemical Engineering & Applied Chemistry, Toronto, ON, Canada

## Abstract

**Background:**

Bacterial membrane vesicles (MVs) are bilipid nanoparticles secreted as a conserved method of intercellular communication. MVs from Gram-positive bacteria carry diverse bacterial products and represent a unique opportunity in the growing field of postbiotics. This native machinery for mediating microbe-host interactions makes MVs a promising tool for intestinal drug delivery in the context of Crohn’s disease (CD). CD is a chronic inflammatory bowel disease characterized by relapsing inflammation of the digestive tract. CD is associated with (1) loss of function mutations in the host gene encoding the nuclear-binding oligomerization domain 2 (NOD2) pattern recognition receptor and (2) a gut microbiome with reduced capabilities to generate NOD2 stimulating muropeptides from peptidoglycan. *Nod2*^*-/-*^ mice have decreased barrier integrity and impaired epithelial restitution upon challenge. However, there are currently no therapeutic strategies targeting NOD2 for CD management.

**Aims:**

The aims of this study were to (1) leverage MVs as a biological system for NOD2 ligand delivery to intestinal epithelial cells and (2) determine the roll of probiotic MVs and bacterial ligand synergy in wound re-epithelialization.

**Methods:**

MVs were purified from *B. subtilis* through filtration and ultracentrifugation. MVs were characterized through nanoparticle tracking analysis using NanoSight300. WT and *NOD2*^*-/-*^ HCT116 cells, a human colorectal carcinoma cell line, were utilized for IL-8 secretion quantification by ELISA, gene expression by qPCR, and *in vitro* scratch wound assay by Incucyte quantification.

**Results:**

Here, we demonstrate for the first time that MVs from *B. subtilis* deliver NOD2 ligands *in vitro*. Treatment with isolated MVs induces IL8 secretion and *CXCL1* expression in a NOD2-dependent manner. Furthermore, MVs promote re-epithelialization after *in vitro* scratch wounding. This effect was completely blunted by the addition of an inhibitor for RIPK2, a downstream transducer required for NOD2 signaling. Through bacterial pathogen-associated molecular patterns (PAMPs) screening, we found that wound re-epithelialization is promoted by PAMP synergy but dependent on RIPK2 signaling.

**Conclusions:**

This work suggests that delivery of muropeptides by probiotic-derived MVs is a promising strategy to promote epithelial regeneration during injury through targeting NOD2. Future directions will work towards validating these findings in human ileal primary cells and murine models of intestinal injury.

**Funding Agencies:**

CIHRMedicine by Design

